# Relationship Between Blood Cytokine Levels, Psychological Comorbidity, and Widespreadness of Pain in Chronic Pelvic Pain

**DOI:** 10.3389/fpsyt.2021.651083

**Published:** 2021-06-25

**Authors:** Bianka Karshikoff, Katherine T. Martucci, Sean Mackey

**Affiliations:** ^1^Department of Clinical Neuroscience, Karolinska Institute, Solna, Sweden; ^2^Department of Anesthesiology, Duke University School of Medicine, Durham, NC, United States; ^3^Division of Pain Medicine, Department of Anesthesiology, Perioperative and Pain Medicine, Stanford University School of Medicine, Palo Alto, CA, United States

**Keywords:** chronic pain, pelvic pain, cytokine-immunological terms, inflammation, comorbidity

## Abstract

**Background:** Low-grade inflammation has been implicated in the etiology of depression, long-term fatigue and chronic pain. TNFα and IL-6 are perhaps the most studied pro-inflammatory cytokines in the field of psychoneuroimmunology. The purpose of our study was to further investigate these relationships in patients with chronic pelvic pain specifically. Using plasma samples from a large, well-described cohort of patients with pelvic pain and healthy controls via the Multidisciplinary Approach to the Study of Chronic Pelvic Pain (MAPP) Research Network, we examined the relationship between TNFα and IL-6 and comorbid psychological symptoms. We also investigated the relationship between IL-8 and GM-CSF, and widespreadness of pain.

**Methods:** We included baseline blood samples in the analyses, 261 patients (148 women) and 110 healthy controls (74 women). Fourteen pro- and anti-inflammatory or regulatory cytokines were analyzed in a Luminex^®^ xMAP^®^ high-sensitivity assay. We used regression models that accounted for known factors associated with the outcome variables to determine the relationship between cytokine levels and clinical measures.

**Results:** There were no statistical differences in cytokine levels between patients and healthy controls when controlling for age. In patients, TNFα was significantly associated with levels of fatigue (*p* = 0.026), but not with pain intensity or depression. IL-6 was not significantly related to any of the outcome variables. Women with pelvic pain showed a negative relationship between IL-8 and widespreadness of pain, while men did not (*p* = 0.003). For both sexes, GM-CSF was positively related to widespreadness of pain (*p* = 0.039).

**Conclusion:** Our results do not suggest low-grade systemic inflammation in chronic pelvic pain. Higher TNFα blood levels were related to higher fatigue ratings, while higher systemic GM-CSF levels predicted more widespread pain. Our study further suggests a potentially protective role of IL-8 with regard to with regard to the widepreadness of pain in the body, at least for women.

## Introduction

An estimated 50–100 million United States (US) adults suffer from chronic pain (CP) with an annual cost of over $500 billion per year, representing one of the most prevalent, costly, and disabling health conditions (1, 2). The highest-need and most impacted patients are those with high-impact chronic pain (affecting ~20 million Americans), or pain associated with substantially restricted work, social, and self-care activities for six or more months (1, 3). Current pharmacological, interventional, behavioral, and surgical therapies for chronic pain are limited in their effectiveness (2, 3). Indeed, chronic pain—and high-impact chronic pain in particular—is often treated with prescription opioids, and is linked to opioid-use disorder. A target for future safe and effective pain treatments may in fact be the immune system and its interaction with the nervous system (4). Experimental and epidemiological studies suggest that the immune system communicates with the central nervous system. It is well-known, for example, that inflammation affects peripheral nerves by sensitizing nerve endings and increasing sensitivity to nociceptive stimuli (5, 6). Psychoneuroimmunological and immunopsychiatric research has expanded the knowledge of neuroimmune interactions, showing that inflammatory components of the immune system also interact with neurons and glial cells in the spinal cord (7, 8), and in the brain (9, 10). Some neuroimmune effects are intrinsically pathological, such as misdirected antibody attacks in rheumatoid arthritis (11) and multiple sclerosis (12). Similar pathological effects potentially trigger schizophrenic episodes over time (13). In contrast, some neuroimmune interactions are adaptive in healthy states (7, 8).

The term “sickness behavior” is often used to describe the behavioral, emotional and physiological adaptations of an organism to the invasion of pathogens (4, 14, 15). These physiological adaptations are driven by inflammatory components secreted by the immune system, and promote recovery during short-term infections. However, some of these adaptations, when dysregulated, appear to lead to complex and persistent illness, such as depressive states (16) and long-term fatigue (17, 18). The role of dysregulated neuroimmune functions in the development and maintenance of chronic pain states is well-documented on a peripheral and spinal level (5, 6, 19, 20). Chronic pain has been associated with elevated blood cytokine levels repeatedly (21), but studies are contradictory. Some cytokines are implicated in several pain disorders, while others seem more specific for a certain syndrome. We and others have suggested that the immune-driven changes, in brain function and affective states, fit mechanistically into the broader understanding of chronic pain (4). Chronic pain is particularly complex, with both physiological and psychological components. To understand how neuroimmune interactions contribute to chronic pain, it is essential to study not only how these contribute to pain intensity, but to the broader experience of pain among patients.

In chronic pain, the brain's generation of the experience of pain may, or may not be, rooted in actual physiological damage. It is believed that as acute pain transitions to chronic states, the nervous system transitions to a state in which the nervous system itself maintains the pain independently from any acute injury. In this chronic state, the pain is very challenging to effectively treat. Several mechanisms that lead to and maintain chronic pain have been suggested, of neurological, psychological and inflammatory nature (4, 22). Neurologically, peripheral and central neurons become hypersensitized, so that the neurons produce greater responses to external stimuli. Neuroimmune mechanisms are involved in this process, linking the immune system to the pain system (5). Researchers have identified sex differences in neuroimmune interactions (19, 20, 23). For example, male rodents require spinal microglial activation, while female rodents appear to use T-cell mediated activity for pain progression. For a review of peripheral and central neuroimmune mechanisms, please see (5, 6, 19, 20). Imaging studies have revealed that the brain processes pain differently once pain has become chronic. Both functional and structural spinal cord and brain changes have been identified in individuals with chronic pain vs. healthy individuals (24–28). Further, these differences in brain structure and function suggest that the brain processes chronic pain differently than acute pain [for an overview see (22, 29–31)]. Some of the areas of identified changes in chronic pain include brain regions involved in emotional process, linking pain to other psychological process, such as anxiety, catastrophizing and depression (26, 32). This indicates that the pain experience, from a subjective and emotional point of view, changes as the pain becomes inescapable and persistent for the afflicted individuals. Conversely, the presence of chronic pain itself increases risk of developing, or worsening, psychiatric problems, creating a vicious circle once the chronic pain state takes hold. Similarly, from a neuroimmune perspective, overlapping neuroimmune influences on the relationship between pain and depression have also been suggested (33, 34). Further, epidemiological and clinical studies show that prior anxious and depressed tendencies, and stress, are risk factors for developing chronic pain (35–39). Few pain syndromes can be explained, or treated, targeting a single mechanism. Peripheral low-grade inflammatory activity may be one of the mechanisms involved in the complexity of chronic pain mechanisms (21). Large-scale peptide analyses suggest ongoing inflammatory activity both centrally and peripherally (40–42) in chronic pain populations. In some studies, the levels correlate with clinical assessments, but not in all, and the focus is often on pain intensity, as this is what one primarily would like to decrease and control. Adding to the challenges, reported cytokines vary between different types of chronic pain and are sometimes even inconsistent across research studies of the same chronic pain condition.

Interestingly, inflammation affects many brain areas that are also involved in pain processing and chronic pain states (9, 10, 43, 44). For example, the insula, the anterior cingulate cortex, amygdala and prefrontal cortices are involved in pain processing and are activated during inflammation. Inflammation has also been shown to directly affect mood, and pain sensitivity in acute inflammatory models. In these models, increased anxiety, depressed mood, and fatigue occur during immune activation (45–47). During immune activation the inflammatory effects are of peripheral origin, but the inflammatory signals are transmitted to the central nervous system (7). These inflammatory signals are transmitted to the brain in a controlled manner via a few dedicated pathways (8). These pathways include the passive transfer of cytokines via brain regions that lack the blood-brain-barrier (BBB), active transport and signaling across the BBB, as well as direct neural signaling, such as via the vagus nerve (14). To improve our understanding of the role of inflammation in chronic pain, we explored the relationship between low-grade inflammation, pain intensity, and psychological measures in patients with chronic pelvic pain. We based our study on hypotheses generated from research on experimental inflammatory models and epidemiological research on populations with inflammatory disease. We had access to a unique sample of blood plasma from chronic pelvic pain patients via the Multidisciplinary Approach to the Study of Chronic Pelvic Pain (MAPP) Research Network and the National Institute of Diabetes and Digestive and Kidney Disease (NIDDK) (48, 49). Standardized sampling procedures ensured high quality plasma samples from a large and well-characterized population of patients with pelvic pain and healthy controls (49). The exact mechanisms that contribute to chronic pelvic pain are still poorly understood, and the MAPP Research Network plays an integral part in advancing understanding of chronic pelvic pain. Historically, chronic pelvic pain has not been largely considered an inflammatory pain disorder. Overall, the population is characterized by a large variation in comorbid pain diagnoses, how widespread the pain is, and psychological comorbidity, as is generally seen in chronic pain populations (28, 50, 51). Prior studies from the MAPP Research Network point to similar levels of pain intensity and psychological comorbidity between men and women with chronic pelvic pain, but more widespread distribution of pain across the body and more comorbid pain diagnoses amongst women (52). Disease severity has been associated with urine markers, such as matrix metalloproteinase (MMP)-2, MMP-9, Lipocalin 2 and vascular endothelial growth factor (VEGF) (53). Regarding blood measures, one prior study showed higher IL-6 levels, but no association with clinical measures, in a subsample of 58 women with chronic pelvic pain (54). However, levels of immunoreactivity, that is, the levels of pro-inflammatory cytokines expressed by immunologically provoked white blood cells, predicted worse outcomes and more pain (54, 55) in women with chronic pelvic pain.

Our primary goal in this cross-sectional study was to assess a broad range of peripheral inflammatory cytokines and characterize cytokine patterns in chronic pelvic pain. Our secondary goal was to characterize the relationship of blood levels of cytokines with pain intensity, widespreadness of pain, and psychological measures in chronic pelvic pain. We hypothesized that inflammatory effects would be more subtle than the well-established variables important for pain, such as stress or anxiety. Yet, inflammatory activity may contribute to biological and psychological aspects of chronic pain, not only affecting the neurons directly, but also impacting psychological well-being in a broader sense. We hypothesized that the pelvic pain group would have higher inflammatory levels in the blood compared to healthy controls (54), and that women in the pain group would have higher inflammatory cytokine levels than men. Furthermore, we hypothesized that TNFα and IL-6 levels would correlate with depressive mood (47, 56, 57) and fatigue (17, 45, 58), when controlling for other psychological factors that are well-established covariates for pain, but not with pain intensity (54, 55). We further hypothesized that higher IL-8 levels would predict more widespread pain, because higher IL-8 levels, both centrally and peripherally, are implicated in several studies of the prototypical widespread pain disorder, fibromyalgia (59–61). As previous MAPP research found that TLR-4 inflammatory response was associated with widespread pain (55), we also performed exploratory analyses on the relationship of the other cytokines in our panel and the widespreadness of pain.

## Methods

### Study Design

The MAPP Research Network is a unique project studying chronic pelvic pain using both subjective (e.g., self-reported survey data) and objective, or quantitative, measures (e.g., neuroimaging scans, blood biomarkers) (49). The project has collected data from 424 (233 women, 191 men) individuals with chronic pelvic pain, 415 healthy controls and 200 patient controls (e.g., irritable bowel syndrome, fibromyalgia, chronic fatigue syndrome). Blood samples, surveys and neuroimaging data were collected at up to four timepoints. For study and sampling details, please see (49, 62).

### Participants

From the whole MAPP sample, participants were primarily included for plasma analysis based on the presence of neuroimaging data and timing of blood draw with neuroimaging data (used for other analyses). In total, 304 (166 f, 138 m) samples from patients with chronic pelvic pain and 110 (74 f, 36 m) healthy control samples were analyzed for plasma cytokines (Table 1). All of these individuals are included in the analyses that compare the chronic pelvic pain group with the healthy controls. However, all surveys were not included at all scanning time points, which is why the number of responses vary for the surveys in Table 2. For the regression analyses exploring the relationship between cytokine levels and subjective ratings, only patients that sampled blood at baseline are included (261 total, 148 f, 113 m), as this is when the pain intensity and stress ratings were matched with the blood sample, to ensure timely matching between ratings and blood markers.

**Table 1:** Median cytokine levels of the pain group vs. the control group.

	**Pelvic pain group**	**Healthy controls**		
**Variable**	**median (IQR)**	**median (IQR)**	***P***	***P* adjusted for age**
GM-CSF	34.38 (20.94–58.06)	36.72 (24.34–69.88)	0.064	0.314
IFNγ	7.06 (4.13–10.87)	7.80 (4.29–10.66)	0.562	0.464
IL-1β	2.06 (1.41–3.36)	2.16 (1.36–3.60)	0.568	0.550
IL-2	2.4 (1.44–3.82)	2.35 (1.59–4.56)	0.244	0.314
IL-4	11.39 (8.28–15.20)	12.34 (8.58–16.71)	0.075	0.149
IL-5	1.13 (0.77–1.43)	1.23 (0.88–1.63)	0.028	0.089
IL-6	0.87 (0.61–1.25)	0.83 (0.63–1.19)	0.860	0.834
IL-8	3.98 (3.17–5.13)	3.98 (3.04–4.99)	0.380	0.959
IL-10	3.14 (2.06–4.55)	3.25 (2.31–4.39)	0.313	0.743
IL-12 (p70)	1.58 (1.05–2.42)	1.74 (1.07–2.54)	0.455	0.702
IL-13	2.50 (1.57–3.54)	2.63 (1.63–3.87)	0.592	0.806
IL-17A	9.88 (5.74–13.92)	10.25 (6.1–14.69)	0.325	0.331
IL-23	137.83 (87.02–289.14)	184.35 (99.56–325.58)	0.095	0.272
TNFα	4.14 (3.35–5.25)	3.81 (3.26–4.58)	0.024	0.216

*Interquartile range and p-value for Mann-Whitney U-test presented*.

*IL, interleukin; GM-CSF, granulocyte-macrophage colony-stimulating factor; IFN, interferon; TNF, tumor necrosis factor*.

**Table 2:** Overview of the covariates of interest in the two groups (mean and standard deviation), and proportion of patients with comorbid pain diagnoses (%).

	**Pelvic pain group**	**Healthy controls**
**Variable**	**Mean (SD)**	**Mean (SD)**
Age	43.47 (14.89)	36.64 (11.45)
BMI	26.33 (5.55)	25.2 (5.02)
Anxiety	7.44 (4.52)	3.52 (2.85)
Depression	5.13 (4.10)	1.47 (2.24)
Fatigue	18.63 (5.42)	12.58 (3.68)
Stress	15.98 (8.03)	9.59 (5.96)
Pain intensity	3.82 (1.9)	0.17 (0.39)
Number of pain sites	5.54 (6.36)	0.69 (1.3)
Comorbid pain diagnoses^a^	55.1 %	

a *Collected in the MAPP study: Fibromyalgia, irritable bowel syndrome, migraine, chronic fatigue, vulvodynia, temporomandibular joint dysfunction. Please see references (49) for more information and deeper analysis*.

### Subjective Ratings

Differences in subjective ratings between groups have been reported and discussed thoroughly before (50–52, 63, 64) and are thus not discussed in this study. Survey measures include The Brief Pain Inventory (BPI) Pain Severity Scores (65, 66), the Hospital Anxiety and Depression Scale (HADS) (67), NIH Patient Reported Outcomes Measurement Information System (PROMIS) Fatigue (7-items) Score (68), the 10-item Perceived Stress Scale (PSS) (69), and the adapted BPI body map for numbers of pain sites (70) [for further details see (49)].

### Cytokine Analysis

All samples were collected at the same time in the morning at nine different study sites, and handled and shipped in a standardized manner (49, 62, 71). The samples were not fasting samples. The Human High Sensitivity T-Cell Discovery Array 14-plex Luminex^®^ xMAP^®^ assay (Millipore MILLIPLEX, Eve Technologies Corp, Calgary, AB, Canada) was used for cytokine analysis (single samples). The cytokines included in the assay were granulocyte-macrophage colony-stimulating factor (GM-CSF), interferon gamma, Interleukin (IL)-1beta, IL-2, IL-4, IL-5, IL-6, IL-8, IL-10, IL-12p70, IL-13, IL-17A, IL-23 and tumor necrosis factor (TNF) α. For sensitivity and accuracy measures, see Supplementary Table 1.

### Statistical Analysis

Mean values for cytokines, age and BMI were calculated. A Mann-Whitney significance test was performed for the cytokines due to non-normal distribution (IBM SPSS Statistics version 25). When controlling for age and BMI, regression analysis and the log-transformed cytokine values (see below) were used.

In order to determine the relationship between cytokine levels and clinical measures, we used regression models that accounted for known factors associated with the outcome variables: age, sex, BMI, pain intensity, stress, anxiety, depression, and fatigue (adapted for the respective outcome variable). For all cytokines, the log-transformed values are used to normalize the distribution. A generalized linear mixed model with log gamma models was chosen to account for clustering of chronic pelvic pain patients within sites, skewed distribution, and heteroscedastic errors. Dependent variables were pain intensity, depression, and fatigue. In these analyses, extreme values were not excluded, but the regression models were performed with and without extremes. The results from the regression models were visually inspected to ensure that the relationships were not driven by one or a few participants.

## Results

The mean values of TNF-a and IL-5 differed between groups, but these differences were not statistically significant when adjusting for age, which differed between the patients with chronic pelvic pain and the healthy controls (Table 1). Adjusting for BMI and sex, which also differed between groups, had no effect on the outcome. Mean values of the variables of interest are presented in Table 2. Spearman correlation for the cytokines are presented in Supplementary Table 2.

In the patient group, TNFα was significantly associated with levels of fatigue (0.22 [0.03 0.41] *p* = 0.026), but not with pain intensity, depression, or widespreadness of pain in the full regression model (Table 3). Sex was significantly associated with fatigue, however, the added interaction term sex × TNFα was not significant. Thus, sex differences were not implicated in the relationship between TNFα levels and fatigue (i.e., how fatigued the patient felt as the time of blood draw). IL-6 was not associated with any of the dependent variables.

**Table 3:** Relationship between TNFα and IL-6, respectively, to fatigue, depression and pain intensity.

**Fatigue**	**B (CI)**	***P***	**Depression**	**B (CI)**	***P***	**Pain intensity**	**B (CI)**	***P***
**TNFα**			**TNFα**			**TNFα**		
(Intercept)	2.17 (1.97 2.38)	<0.001	(Intercept)	−0.33 (−0.77 0.70)	0.929	(Intercept)	0.78 (0.28 1.28)	0.002
Sex	0.12 (0.07 0.18)	<0.001	Sex	−0.39 (−0.58 −0.19)	<0.001	Sex	0.08 (−0.06 0.21)	0.240
Age	0 (0)	0.351	Age	0.01 (0 0.01)	0.082	Age	0 (−0.01 0)	0.192
BMI	0 (0)	0.160	BMI	−0.01 (−0.02 0)	0.285	BMI	0.01 (−0.01 0.02)	0.314
Anxiety	0 (0)	0.396	Anxiety	0.02 (0.01 0.05)	0.208	Anxiety	−0.01 (−0.03 0.02)	0.501
Depression	0.02 (0.01 0.03)	<0.001	Fatigue	0.05 (0.03 0.08)	<0.001	Depression	0.05 (0.02 0.07)	0.000
Stress	0.01 (0.01 0.02)	<0.001	Stress	0.04 (0.02 0.06)	<0.001	Fatigue	0.02 (0 0.03)	0.034
Pain intensity	0.02 (0.00 0.04)	0.018	Pain intensity	0.07 (0.02 0.12)	0.008	Stress	0 (−0.02 0.01)	0.661
TNFα	0.22 (0.03 0.41)	0.026	TNFα	−0.02 (−0.74 0.70)	0.960	TNFα	−0.09 (−0.57 0.40)	0.722
**IL-6**			**IL-6**			**IL-6**		
(Intercept)	2.32 (2.13 2.52)	<0.001	(Intercept)	−0.19 (−0.86 0.48)	0.578	(Intercept)	0.75 (0.32 1.18)	0.001
Sex	0.12 (0.07 0.18)	<0.001	Sex	−0.40 (−0.59 −0.20)	<0.001	Sex	0.08 (−0.05 0.21)	0.225
Age	0 (0)	0.473	Age	0.01 (0 0.01)	0.060	Age	0 (−0.01 0)	0.188
BMI	0 (0)	0.082	BMI	−0.01 (−0.03 0.01)	0.250	BMI	0.01 (−0.01 0.02)	0.333
Anxiety	0 (0)	0.401	Anxiety	0.02 (−0.01 0.05)	0.201	Anxiety	−0.01 (−0.32 0.14)	0.455
Depression	0.02 (0.01 0.03)	<0.001	Fatigue	0.05 (0.03 0.08)	<0.001	Depression	0.05 (0.02 0.07)	0.000
Stress	0.01 (0.01 0.02)	<0.001	Stress	0.04 (0.02 0.06)	<0.001	Fatigue	0.02 (0 0.03)	0.050
Pain intensity	0.02 (0.00 0.03)	0.038	Pain intensity	0.08 (0.02 0.13)	0.005	Stress	0 (−0.02 0.01)	0.730
IL-6	−0.03 (−0.08 0.02)	0.250	IL-6	0.12 (−0.04 0.28)	0.146	IL-6	−0.14 (−0.42 0.14)	0.327

*Means, 95% confidence intervals and p-values for the models including TNFα and IL-6 presented*.

*The estimates represent the change in relation to log transformed values of the cytokine analysis*.

**Figure 1:**
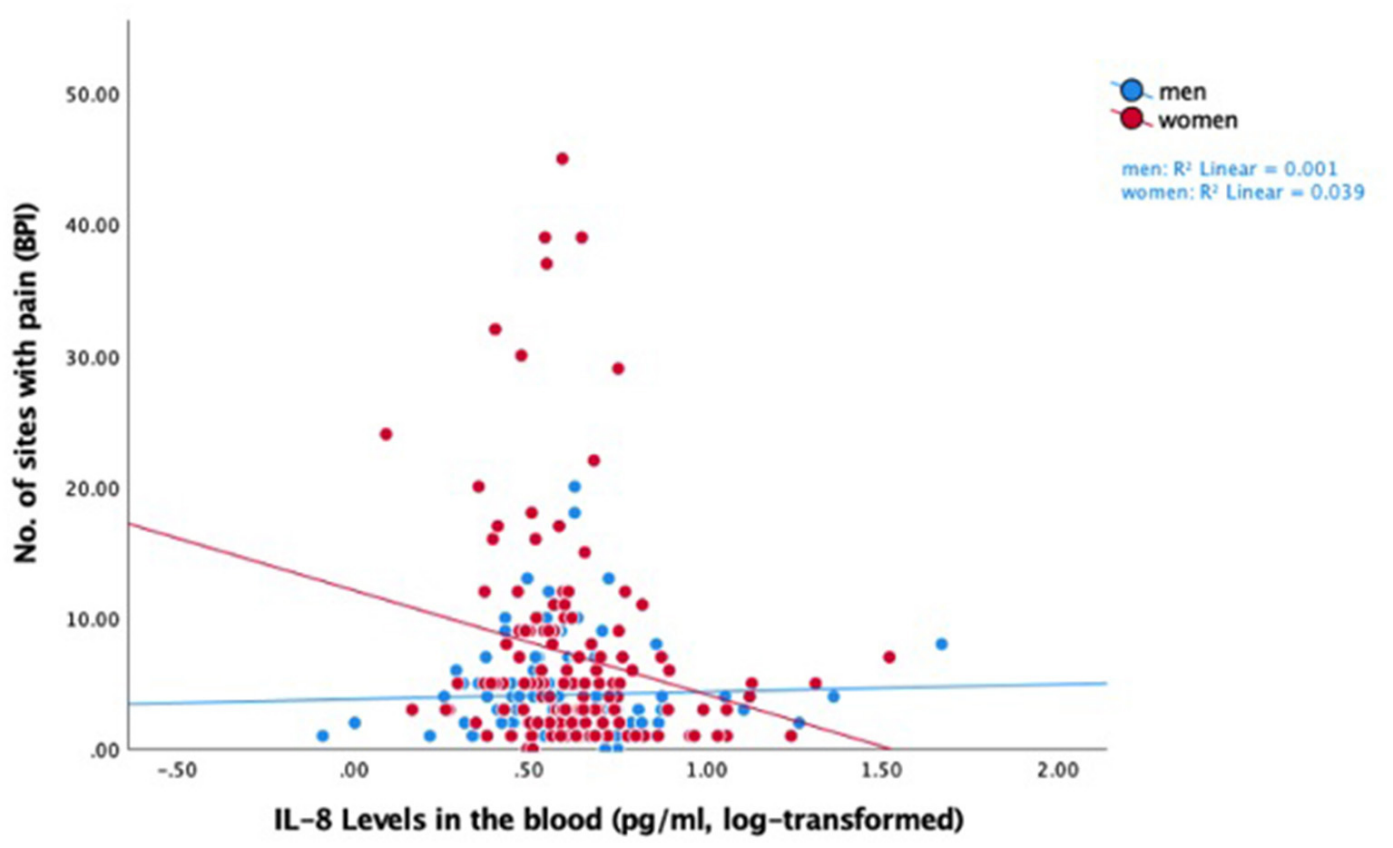
Raw data scatter plot of the relationship between log-transformed IL-8 levels and widespreadness of pain, for men and women in the pelvic pain group.

IL-8 was showed a negative relationship with widespreadness of pain, that is, lower IL-8 levels were associated with more pain sites across the body (−0.44 [−0.87 −0.02] *p* = 0.039). This relationship was driven only by women with chronic pelvic pain (−1.30 [−2.14 −0.045] *p* = 0.003, Table 4 and Figure 1).

**Table 4:** Relationship between IL-8 and GM-CSF and the widespreadedness of pain in the patient group.

**No. of sites with pain**	**B (CI)**	***P***
*IL-8*
(Intercept)	−1.14 (−2.19 to 0.08)	0.036
Sex	1.06 (0.5 to 1.6)	<0.001
Age	0 (−0.01 to 0)	0.399
BMI	0.01 (−0.0to 0.03)	0.177
Anxiety	0.02 (0.02 to 0.05)	0.432
Depression	0 (−0.03 to 0.04)	0.890
Fatigue	0.06 (0.03 to 0.08)	<0.001
Stress	0 (−0.02 to 0.03)	0.909
Pain intensity	0.03 (−0.3 to 0.09)	0.355
IL-8	1.44 (0.09 to 2.78)	0.036
Sex ^*^ IL-8	−1.30 (−2.14 to 0.45)	0.003
*GM-CSF*
(Intercept)	0.31 (−0.58 to 1.20)	0.493
Sex	0.34 (0.12 to 0.57)	0.004
Age	0 (−0.01 to 0)	0.283
BMI	0.01 (−0.01 to 0.03)	0.274
Anxiety	0 (−0.04 to 0.04)	0.961
Depression	0.01 (−0.03 to 0.05)	0.586
Fatigue	0.05 (0.02 to 0.08)	<0.001
Stress	0.01 (−0.01 to 0.04)	0.610
Pain intensity	0.02 (−0.04 to 0.08)	0.460
GM-CSF	−0.36 (−0.70 to 0.02)	0.039

*Means, 95% confidence intervals and p-values for the models including IL-8 and GM-CSF presented. The estimates represent the change in relation to log-transformed values of the cytokine analysis*.

In the explorative analyses, GM-CSF showed a positive relationship with the widespreadness of pain (i.e., number of pain sites across the body) (−0.36 [−0.70 −0,02] *p* = 0.039) (Table 4), suggesting that higher GM-CSF levels predicted more widespread pain. The association between GM-CSF and widespreadness of pain was not sex dependent.

### Robustness of the Regression Models

Simple models with only sex, age, and BMI as covariates did not show an association between TNFα and fatigue, or between GM-CSF and number of pain sites, however the significant findings for IL-8 and widespreadness of pain remained (Supplementary Table 3). Models adding medicine usage at the time of the lab visit and duration of pain in years, variables that may in theory also be related to the investigated outcome variables, did not improve the model fit or change the results in any significant way. As these analyses were exploratory, we did not control for multiple comparisons.

## Discussion

Our aim was to characterize cytokine blood levels in patients with chronic pelvic pain and relate cytokine levels to clinical characteristics. We used a high-sensitivity cytokine panel, as it is known that cytokine levels in patients with chronic pain resemble the levels in healthy individuals, as compared to acutely inflamed patient groups, such as cancer patients. The 14 pro- and anti-inflammatory cytokines in the panel have been implicated previously in chronic pain research. We could not show a difference in any of the analyzed systemic cytokines between patient and healthy controls, when controlling for age and BMI. Prior findings from the Research Network reported higher IL-6 levels in chronic pelvic pain compared to healthy controls (54) in a subgroup of women with pelvic pain, which was not replicated in this larger group including both sexes. Overall, our results suggest that this patient population does not seem to suffer from an ongoing low-grade inflammation. However, in the patient group, we saw a positive relationship between TNFα levels in the blood and subjectively rated fatigue levels. Higher TNFα levels were associated with more fatigue in both men and women with chronic pelvic pain. TNFα-levels were not related to depression scores or pain intensity, nor was IL-6.

Low or normal blood levels may not be the crucial point when looking into relationships between immune markers and clinical aspects in chronic pain. Theoretically, low levels of circulating signaling molecules could induce a stronger response in a primed immune system. Immunoreactivity can be assessed by provoking cytokine-producing immune cells in some way, for example, by inflammatory stimuli such as lipopolysaccharides, or stress stimuli such as glucocorticoids, or specific receptor ligands. The provoked response shows how reactive, or primed, the immune system is, despite low-grade systemic activity under normal circumstances. From the MAPP initiative, there is evidence that immunoreactivity is enhanced in individuals with chronic pelvic pain, at least in women (54, 55, 72), and that this exaggerated immunoreactivity is associated with comorbid symptoms and the lack of disease resolution over time. TNFα levels in chronic pelvic pain patients may be specifically important for the feeling of fatigue. For patients with chronic pain, the profound fatigue that many experience can be almost as debilitating at the pain itself (73). Patients describe the fatigue as different and more pronounced than normal fatigue, and with stronger cognitive effects than fatigue experienced under healthy circumstances (17, 74). In individuals with rheumatoid arthritis, anti-cytokine treatments are effective for fatigue, in addition to pain-relieving and anti-inflammatory benefits (75). However, over time, long-term fatigue often persists even when the rheumatoid arthritis swelling and pain are under control (76). Mechanistically, inflammation-driven fatigue is yet to be fully explained (17), but the phenomenon is increasingly being recognized, and several potential pathways have been suggested. These potential pathways of inflammation-driven fatigue include mechanisms of oxidative stress and mitochondrial dysfunction (77), as well as an imbalance in energy availability and expenditure driven by inflammation-induced insulin resistance (74). However, it is important to note that these mechanisms are rather distinct from potential pathways of inflammation-driven pain.

In contrast, for chronic pain and depression, several common neuroinflammatory pathways have been proposed (33, 34). One of the most interesting pathways with regard to both pain and depression, may be the kynurenine and tetrahydrobiopterin (BH4) pathways (78, 79). BH4 blockers show promising treatment abilities with few side effects for pain (80). These interacting pathways are readily affected by peripheral inflammation, and they limit the availability of several monoamines, including serotonin. In pain research, the main location of study of immune-to-CNS connections is the spinal cord, while in depression research, the study of immune-to-CNS connections is focused on the brain. It is not known how the crosstalk and regulation between spinal cord and brain areas occur for neuroimmune communication. However, for pain processing, the communication between spinal cord and brain is constant and bi-directional. Despite neuroimmune overlap between pain and depression, our findings did not suggest a relationship between pro-inflammatory blood levels and depressive states in chronic pelvic pain. It should be noted that none of the patients suffered from clinical depression. Only ten individuals had HADS scores of 11 or higher, which would be the cut-off for suspected clinical depression (67, 81). In experimental inflammatory models, depressed mood is readily induced by immune activation (14, 15). In these models however, the peak blood cytokine levels are higher than in the chronic pelvic pain population. With regard to clinical depression, the connection to inflammatory activity is undisputable (56, 57, 82, 83), but the relationship is complex and potentially only relevant for a subgroup of clinically depressed patients. For the studied patient group of non-inflammatory chronic pelvic pain and relative psychological health, immune-driven depressive mood may not be a major concern.

In individuals with chronic pain, an important clinical feature is where pain is felt across the body. In general, as the distribution of experienced pain is more widespread across the body of an individual, this can contribute to greater suffering and greater challenges in finding successful treatment options. Fibromyalgia is a chronic pain condition characterized by widespread pain across the body. Several studies of fibromyalgia implicate a role for IL-8, a pro-inflammatory cytokine (59–61). We therefore aimed at understanding the relationship of IL-8 and widespreadness of pain in our cohort of individuals with chronic pelvic pain. Among the women with chronic pain in our study cohort, we did see a relationship between IL-8 levels and number of painful body sites. However, the relationship was opposite to our hypotheses, with lower IL-8 levels peripherally related to greater widespreadness of pain. This suggests that IL-8 could have a protective role in chronic pelvic pain, at least in women, and in pain conditions that are not characterized by high peripheral inflammatory activity. We are not aware of any studies that report such an inverted and sex-dependent relationship for blood IL-8 levels, but a previous study in osteoarthritis patients reported a negative relationship for CSF IL-8 levels with both physical function and quality of life measures (84). On the contrary, GM-CSF levels appeared to have a non-protective, pain exacerbating role, with lower GM-CSF predicting less widespreadness of pain in our chronic pelvic pain population. This correlation was not sex dependent. GM-CSF is an immunoregulatory cytokine that can tap into pro- and anti-inflammatory pathways in feedback loops and has a key role in homoeostasis and pathogen clearance. However, GM-CSF has been implicated in several inflammatory and autoimmune disorders. GM-CSF is believed to play a critical role in both the resolution of inflammatory responses, as in the development of chronic inflammation [for an overview see (85–87)]. With regard to pain, the actions of the GM-CSF cytokine appear somewhat contradictory. Anti GM-CSF treatment seems promising in inflammatory pain states, such as rheumatoid arthritis and osteoarthritis (88). For example, in an osteoarthritis model, GM-CSF deficient mice develop less inflammatory pain than wildtype mice (89). In contrast, lower amounts of GM-CSF expressing cells in the synovial lining are associated with greater knee pain intensity in patients with osteoarthritis (90). Additionally, lower blood levels of GM-CSF have been identified in chronic back patients compared to healthy controls (91). Teware et al. argue that while nociceptors themselves may have receptors for GM-CSF, GM-CSF may in fact modulate pain perception specifically *via* secondary, inflammatory pathways (92). Seemingly contradictory relationships between GM-CSF and depression have been identified as well. For example, one recent study found elevated GM-CSF levels in adolescent major depression (93), while a recent rodent study found that GM-CSF has anti-depressive effects (94). Ultimately, the roles of IL-8 and GM-CSF in chronic pain require further investigation.

### Limitations and Future Directions

Our study has several limitations. First, we studied a limited set of cytokines with single sample analyses in plasma, and these analyses were selected as a starting point in order to achieve an overall understanding of the peripheral inflammatory state of the MAPP chronic pelvic pain patient cohort. Second, our analyses include several covariates, therefore we acknowledge a risk of overfitting the model. Nonetheless, we believe that the sample is large enough to accommodate our statistical choices. Ethnicity and other demographics were not available in this substudy of MAPP and were not included in the analyses. Furthermore, the estimates are related to log-transformed plasma cytokine levels in pg/ml, which makes them hard to interpret in a practical sense, for example, to estimate the relevance for clinical treatment. The results of this study are thus to be interpreted as potential mechanistic relationships to be further explored.

Our findings exemplify the complexity of studying blood cytokines in patient groups, particularly in cross-sectional studies. In cross-sectional studies, we get a glimpse of the current biological and psychological status of a patient, but we cannot know which “developmental stage” of the chronicity of the disease the individuals are currently experiencing. The biological networks of an organism will adapt during the course of a disease as part of allostasis. Presumably, the feedback loops lose their normal regulatory function over time as the strain on the biological systems becomes long-term or chronic. This is exemplified by the flattening of the hypothalamic-pituitary-adrenal (HPA) axis in many chronic disorders, including pain (95). This means that a higher or lower level of a cytokine may have a different impact on the biological system it governs in the beginning of a disease, than it may have 10 years later. Furthermore, it is important to question the common assumption that increases in pro-inflammatory cytokines, and conversely decreases in anti-inflammatory cytokines, are problematic in chronic disease. Importantly, organisms would not survive without inflammation. In fact, in some aspects of disease development, increases in inflammatory activity appear to be protective. For instance, IL-8 may play a protective role in women with chronic pelvic pain as suggested by our observed relationship between lower IL-8 and greater widespreadness of pain. Finally, different cytokine networks may be responsible for pain intensity and psychological distress, as suggested by a recent study in fibromyalgia (96). A comprehensive understanding of neuroimmune mechanisms in chronic pain will require human longitudinal studies, largescale peptide analyses, and consideration of a large variety of covariates.

## Conclusions

Overall, the population with chronic pelvic pain does not show a pro-inflammatory cytokine profile in the blood, but cytokine levels resemble those of healthy individuals. Higher TNFα blood levels were related to higher fatigue ratings, while higher systemic GM-CSF levels predicted more widespread pain. Widespreadness of pain in women was, contrary to our hypotheses, negatively correlated with IL-8 blood levels, suggesting a potentially protective role of IL-8. None of the studied cytokines correlated with pain intensity. We conclude that when studying inflammatory mechanisms in chronic pain, psychological measures and the spread of pain need to be considered along with pain severity.

## Data Availability Statement

All data supporting the conclusions of this article can be obtained from The National Institute of Diabetes and Digestive and Kidney Diseases (NIDDK) repository. Further enquiries should be directed to the authors.

## Ethics Statement

The studies involving human participants were reviewed and approved by Stanford University. The patients/participants provided their written informed consent to participate in this study.

## Author Contributions

BK: statistical analyses and drafted manuscript. All authors conceptualization and design of the study, interpretation of results, read, modified, and approved the final manuscript.

## Conflict of Interest

The authors declare that the research was conducted in the absence of any commercial or financial relationships that could be construed as a potential conflict of interest. The reviewer MP declared a shared research group with the authors at time of review.
