# Supplementary material for: Relationship Between Blood Cytokine Levels, Psychological Comorbidity, and Widespreadness of Pain in Chronic Pelvic Pain
Source: Front Psychiatry. 2021 Jun 25;12:651083. doi: 10.3389/fpsyt.2021.651083 (PMC8267576; doi:10.3389/fpsyt.2021.651083)
Supplement: Supplementary file 1 [file Table_1.docx]

**Supplementary Table 1:** Sensitivity and accuracy measures for the Human High Sensitivity T-Cell Discovery Array 14-Plex Assay Kit Source: Millipore MILLIPLEX. Source: https://www.evetechnologies.com/product/human-high-sensitivity-t-cell-discovery-array-14-plex/.

| **Cytokine** | **Sensitivity** | **Intra-assay** | **Inter-assay** | **Accuracy** |  |  |
| --- | --- | --- | --- | --- | --- | --- |
| Mo | pg/mL | %CV | %CV | % Recovery | Excluded |  |
| **GM-CSF** | 0,35 | < 5 | < 15 | 101 | 5 |  |
| **IFNγ** | 0,48 | < 5 | < 20 | 101 | 3 |  |
| **IL-1β** | 0,14 | < 5 | < 15 | 98 | 1 |  |
| **IL-2** | 0,19 | < 5 | < 15 | 103 | 2 |  |
| **IL-4** | 1,12 | < 5 | < 15 | 103 | 2 |  |
| **IL-5** | 0,12 | < 5 | < 20 | 101 | 1 |  |
| **IL-6** | 0,11 | < 5 | < 20 | 107 | 1 |  |
| **IL-8** | 0,13 | < 5 | < 15 | 103 | 0 |  |
| **IL-10** | 0,56 | < 5 | < 20 | 104 | 4 |  |
| **IL-12 (p70)** | 0,15 | < 6 | < 15 | 100 | 1 |  |
| **IL-13** | 0,23 | < 5 | < 20 | 103 | 2 |  |
| **IL-17A** | 0,33 | < 5 | < 20 | 106 | 1 |  |
| **IL-23** | 3,25 | < 5 | < 20 | 100 | 0 |  |
| **TNFα** | 0,16 | < 5 | < 15 | 103 | 0 |  |
