# Supplementary material for: Relationship Between Blood Cytokine Levels, Psychological Comorbidity, and Widespreadness of Pain in Chronic Pelvic Pain
Source: Front Psychiatry. 2021 Jun 25;12:651083. doi: 10.3389/fpsyt.2021.651083 (PMC8267576; doi:10.3389/fpsyt.2021.651083)
Supplement: Supplementary file 2 [file Table_2.docx]

|  |  | **1** | **2** | **3** | **4** | **5** | **6** | **7** | **8** | **9** | **10** | **11** | **12** | **13** | **14** |
| --- | --- | --- | --- | --- | --- | --- | --- | --- | --- | --- | --- | --- | --- | --- | --- |
| **1.** | **GMCSF** |  |  |  |  |  |  |  |  |  |  |  |  |  |  |
| **2.** | **IFNg** | .56** |  |  |  |  |  |  |  |  |  |  |  |  |  |
| **3.** | **IL1b** | .27** | .27** |  |  |  |  |  |  |  |  |  |  |  |  |
| **4.** | **IL2** | .60** | .77** | .34** |  |  |  |  |  |  |  |  |  |  |  |
| **5.** | **IL4** | .42** | .51** | .25** | .63** |  |  |  |  |  |  |  |  |  |  |
| **6.** | **IL5** | .55** | .65** | .33** | .70** | .54** |  |  |  |  |  |  |  |  |  |
| **7.** | **IL6** | .36** | .61** | .24** | .59** | .46** | .52** |  |  |  |  |  |  |  |  |
| **8.** | **IL8** | .29** | .43** | .39** | .43** | .31** | .41** | .59** |  |  |  |  |  |  |  |
| **9.** | **IL10** | .47** | .58** | .28** | .63** | .51** | .63** | .55** | .38** |  |  |  |  |  |  |
| **10.** | **IL12p70** | .57** | .75** | .28** | .81** | .54** | .68** | .59** | .37** | .59** |  |  |  |  |  |
| **11.** | **IL13** | .39** | .57** | .12* | .57** | .50** | .55** | .69** | .42** | .57** | .54** |  |  |  |  |
| **12.** | **IL17A** | .57** | .92** | .28** | .79** | .51** | .65** | .63** | .47** | .57** | .76** | .55** |  |  |  |
| **13.** | **IL23** | .56** | .68** | .37** | .76** | .55** | .65** | .58** | .46** | .57** | .72** | .54** | .71** |  |  |
| **14.** | **TNFa** | -.13** | -.06 | .21** | -.05 | -.06 | -.06 | .12** | .32** | .02 | -.07 | -.01 | -.05 | -.04 |  |

**Supplementary Table 2.** Spearman correlation for the cytokines in the whole sample.

** Correlation is significant at the 0.01 level (2-tailed)

* Correlation is significant at the 0.05 level (2-tailed)
