# Supplementary material for: Relationship Between Blood Cytokine Levels, Psychological Comorbidity, and Widespreadness of Pain in Chronic Pelvic Pain
Source: Front Psychiatry. 2021 Jun 25;12:651083. doi: 10.3389/fpsyt.2021.651083 (PMC8267576; doi:10.3389/fpsyt.2021.651083)
Supplement: Supplementary file 3 [file Table_3.docx]

**Supplementary Table 3:** Simple models with only age, sex and BMI as covariates for the models that showed a significant effect of a cytokine in the main analysis.

Means, 95% confidence intervals and p-values presented.

|  |  |  |
| --- | --- | --- |
| **Fatigue** | B (CI) | *P* |
| *TNFα* |  |  |
| (Intercept) | 2.55 (2.30 2.80) | <0.001 |
| Sex | 0.15 (0.77 2.14) | <0.001 |
| Age | -0.01 (-0.01 -0.00) | <0.001 |
| BMI | 0.01 (0.01 7.22) | 0.007 |
| TNFα | 0.18 (-0.07 0.44) | 0.159 |
| **No. of sites with pain** | B (CI) | *P* |
| *IL-8* |  |  |
| (Intercept) | -0.58 (-1.74 0.57) | 0.323 |
| Sex | 1.41 (0.79 2.03) | <0.001 |
| Age | -0.01 (-0.02 -0.00) | 0.012 |
| BMI | 0.03 (0.01 0.05) | 0.001 |
| IL-8 | 1.709 (0.18 3.24) | 0.029 |
| Sex * IL-8 | -1.473 (-2.4 -0.53) | 0.002 |
|  |  |  |
| *GM-CSF* |  |  |
| (Intercept) | 1.02 (0.11 1.94) | 0.029 |
| Sex | 0.57 (0.34 0.79) | <0.001 |
| Age | -0.01 (-0.02 -0.00) | 0.004 |
| BMI | 0.03 (0.01 0.05) | 0.002 |
| GM-CSF | -0.35 (-0.71 0.02) | 0.061 |

*The estimates represent the change in relation to a log-transformed values of the cytokine analysis.*
